# Apoptosis induced by β,β-dimethylacrylshikonin is associated with Bcl-2 and NF-κB in human breast carcinoma MCF-7 cells

**DOI:** 10.3892/ol.2013.1613

**Published:** 2013-10-10

**Authors:** YAO XIONG, XIU-YING MA, ZIRAN ZHANG, ZHEN-JUN SHAO, YUAN-YUAN ZHANG, LI-MING ZHOU

**Affiliations:** 1 Department of Pharmacology, Preclinical and Forensic Medical College, Sichuan University, Chengdu, Sichuan 610041, P.R. China; 2 Hongjitang Pharmaceutical Co., Ltd., Jinan, Shandong 250000, P.R. China

**Keywords:** β,β-dimethylacrylshikonin, breast cancer, apoptosis, nuclear factor-κB

## Abstract

β,β-dimethylacrylshikonin (DA) is a natural naphthoquinone derivative compound of *Lithospermum erythrorhizon* with various biological activities. The present study aimed to investigate the inhibitory effects and underlying mechanisms of DA in human breast carcinoma MCF-7 cells. The 3-(4,5-dimethylthiazol-2-yl)-2,5-diphenyltetrazolium bromide (MTT) assay showed that DA inhibited the proliferation of MCF-7 cells in a dose- and time-dependent manner. The half maximal inhibitory concentration of DA with regard to the proliferation of MCF-7 cells was 0.050±0.016 mM. The characteristics of cell apoptosis, including cell shrinkage, nuclear pyknosis and chromatin condensation, were all observed in DA-treated cells. DA decreased the expression levels of Bcl-2 and increased the expression of Bax and caspase-3 compared with those in the control. DA inhibited the activity of the nuclear factor (NF)-κB pathway, by downregulating the expression of the p65 subunit, and inhibited the Iκb phosphorylation. DA inhibits the proliferation of MCF-7 cells *in vitro* by inducing apoptosis through the downregulation of Bcl-2, upregulation of Bax and partial inactivation of the NF-κB pathway.

## Introduction

Breast carcinoma is the most commonly diagnosed cancer in females of all ethnic groups and its incidence and mortality rank second in China (1). According to Cancer Statistics 2012 (2), breast cancer was the most common form of cancer among females in USA. In the USA, there are ~40,000 female breast cancer mortalities and 230,480 new cases of breast cancer each year. Current therapeutic approaches for human breast cancer include hormonal therapy with anti-estrogenic compounds, as well as surgery, radiotherapy, hyperthermia and chemotherapy (3). At present, patients with breast cancer have certain clinical responses to these strategies, although they remain limited in the clinic. Novel and effective treatments for breast cancer are urgently required.

**Figure 1:**
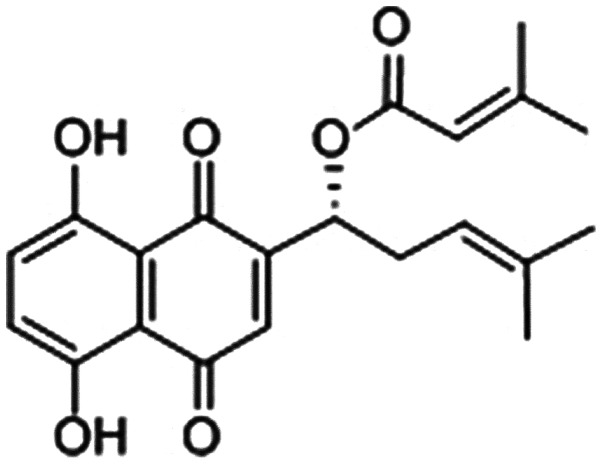
Chemical structure of β,β-dimethylacrylshikonin.

β,β-dimethylacrylshikonin (DA; Fig. 1) is a natural naphthoquinone derivative compound from the root tissues of *Lithospermum erythrorhizon* (*L. erythrorhizon*) a famous Chinese medical herb called ‘Zicao’ (4). *L. erythrorhizon* has been widely used as a traditional Chinese medicine for thousands of years to treat burns or promote wound healing through its antibacterial and anti-inflammatory activities (5). Shikonin and its derivatives have been demonstrated to exert anticancer and apoptotic activities against tumor cells, such as sarcoma 180 (S-180) ascites cells, gastric cancer, hepatocellular carcinoma, colon adenocarcinoma, epidermoid carcinoma, leukemia and prostate cancer (6–10). However, little is known regarding the effects and mechanisms of DA in breast cancer cells.

The present study aimed to evaluate the antitumor effects of DA on the human breast cancer MCF-7 cell line and investigate its molecular mechanisms.

## Materials and methods

### Reagents

DA was obtained from Huakang Pharmaceutical Company (Deyang, China) and the purity was demonstrated to be >98% by high performance liquid chromatography. Primary rabbit anti-human p65, rabbit anti-human Iκb and phosphorylated Iκb antibodies were purchased from Cell Signaling Technology, Inc. (Danvers, MA, USA). All secondary antibodies were obtained from Santa Cruz Biotechnology, Inc. (Santa Cruz, CA, USA).

### Cell culture

MCF-7 cells were obtained from the Shanghai Institute of Cell Biology (Chinese Academy of Sciences, Shanghai, China) and maintained in RPMI-1640 (Hali, Chengdu, China) supplemented with 10% fetal bovine serum (HyClone, Logan, UT, USA), 100 U/ml penicillin and 100 μg/ml streptomycin (Huabei Pharmaceuticals Ltd., Shijiazhuang, China), in a humidified atmosphere with 5% CO_2_ at 37°C.

### 3-(4,5-Dimethylthiazol-2-yl)-2,5-diphenyltetrazolium bromide (MTT) assay

The inhibitory effects of DA on the proliferation of MCF-7 cells were measured using the MTT assay (7). The cells were seeded in 96-well plates at a density of 5×10^3^/well. Following incubation for 24 h, the cells were treated with the indicated concentrations of DA (0.27, 0.135, 0.0675, 0.0337, 0.0169, 0.0084 and 0.0042 mM) for 24, 48 and 72 h. Subsequently, 20 μl MTT (Sigma, St. Louis, MO, USA) solution (5 mg/ml) in phosphate-buffered saline (PBS) was added at 24, 48 and 72 h after treatment, followed by incubation for a further 4 h. After the medium was removed, dark blue formazan was dissolved in 150 μl DMSO (Sigma). Following agitation for 15 min, the optical density of each well was measured with a 680c microplate reader (Bio-Rad, Hercules, CA, USA) at a wavelength of 570 nm. The 50% inhibitory concentration (IC_50_) was determined via the Bliss method (11).

### 4,6-Diamidino-2-phenylindole dihydrochloride hydrate (DAPI) staining

Apoptotic cells were detected using DAPI (Sigma) staining (5), which identifies typical apoptotic nuclear changes, including condensed and fragmented nuclei. The cells were plated in 6-well plates at a density of 2×10^4^/well. After treatment with DA for 48 h, the cells were fixed using PBS containing 4% paraformaldehyde for 30 min and incubated with DAPI (1 mg/ml) for a further 30 min. The cells were then visualized under a fluorescence microscope (DMI4000B; Olympus, Tokyo, Japan) with a 360–370 nm excitation light and a 420–460 nm emission filter.

### Flow cytometry

Annexin V/propidium iodide (PI) staining was employed to detect the morphological changes of the cells (12). After DA treatment, cells were harvested and washed with ice-cold PBS three times. The cells were then stained with Annexin V-FITC and PI, and monitored for apoptosis using flow cytometry according to the manufacturer’s instructions (Boster, Wuhan, China). Non-stained cells were indicated to be viable. PI-positive staining indicated necrosis, while Annexin V-FITC-positive staining showed cells in the early stages of apoptosis. PI- and Annexin V-FITC-positive cells were considered to be in late stage apoptosis. Additional exposure to PI made it possible to differentiate the early apoptotic cells from the late apoptotic ones.

### Reverse-transcription polymerase chain reaction (RT-PCR)

The expression levels of Bcl-2, Bax and caspase-3 mRNA were measured using RT-PCR as previously described (13). MCF-7 cells were plated at a density of 5×10^4^/well into six-well plates for 24 h. The cells were then treated with the indicated concentrations of DA (0.0125, 0.025 and 0.05 mM) for 48 h. Total mRNA was extracted from DA-treated or control cells using TRIzol reagent (Invitrogen, Carlsbad, CA, USA) and reverse transcribed using the RevertAid™ First Strand cDNA Synthesis kit (Fermentas, Burlington, ON, Canada), following the manufacturer’s instructions. The primer sequences used in this study were as follows: Bcl-2, 5′-TGTGGCCTTCTTT GAGTTCG-3′ and 5′-TCACTTGTGGCTCAGATAGG-3′; Bax, 5′-GCGTCCACCCAAGAAGCTGAG-3′ and 5′-ACCAC CCTGGTCTTGGATCC-3′; caspase-3, 5′-CAAACTTTT CAGAGGGGATCG-3′ and 5′-GCATACTGTTTCAGCATGGCAC-3′; β-actin, 5′-TCACCCACACTGTGCCCATC TACGA-3′ and 5′-CAGCGGAACCGCTCATTGCCAA TGG-3′. β-actin was used in each experiment as an internal control. The PCR products were electrophoresed on 1% agarose gels, stained with ethidium bromide and observed under ultraviolet light. The relative mRNA levels were expressed as the ratio of the signal intensity of the target gene to that of β-actin. Analysis was performed with Quantity One V4.62 software (Bio-Rad).

### Western blotting

Proteins from the cell lysates of DA-treated MCF-7 cells were obtained using lysis buffer [50 mmol/l Tris (pH 7.5), 100 mmol/l NaCl, 1 mmol/l EDTA, 0.5% NP40, 0.5% Triton X-100 and 1 mmol/l PMSF] and protein concentrations were measured with a Bio-Rad Protein Assay kit (Bio-Rad) based on the Bradford method, according to the manufacturer’s instructions. Proteins were incubated for 3 min at 100°C prior to electrophoresis, then separated using 12% SDS-PAGE at 120 V for 3–4 h. The proteins were transferred onto polyvinylidene difluoride (PVDF) membranes (Bio-Rad). After blocking with 5% non-fat milk at 4°C overnight, the membranes were incubated in fresh 5% Tris-buffered saline with Tween-20 (TBST)-Bovine lacto transfer technique optimizer with 1:500 primary antibodies for 2 h at room temperature. After being washed with TBST for 10 min, the PVDF membranes were incubated with secondary antibodies for 1 h. Proteins were then detected with the Superstar Enhanced Chemiluminescent kit (AR1111; Boster). The expression levels of the proteins were compared with those of the β-actin control, based on the relative intensities of the bands. Band density was quantified using Quantity One V4.62 software (Bio-Rad).

### Statistical analysis

Data are expressed as the mean ± SD of three independent experiments. The Statistical Package for Social Sciences version 13.0 (SPSS Inc., Chicago, IL, USA) was used for standard statistical analysis by one-way analysis of variance. P<0.05 was considered to indicate a statistically significant difference.

## Results

### DA inhibits the proliferation of MCF-7 cells

**Figure 2:**
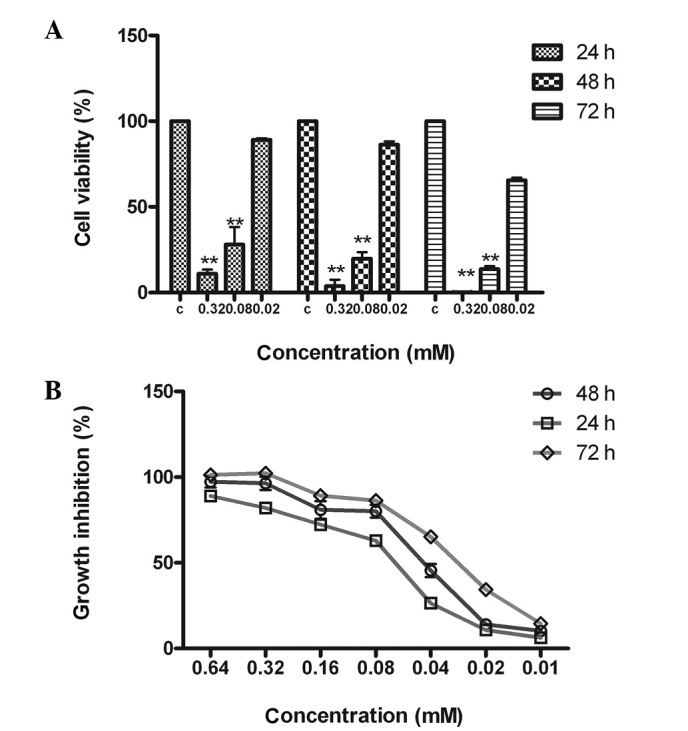
(A and B) DA inhibited the proliferation of MCF-7 cells. Cells were treated with various concentrations of DA for 24, 48 and 72 h. The associated cell viability was determined by MTT assays. DA treatment resulted in a significant dose- and time-dependent decrease in cell proliferation compared with that in untreated cells. Each experiment was independently performed three times. ^**^P<0.01, compared with the control group. DA, β,β-dimethylacrylshikonin; MTT, 3-(4,5-dimethylthiazol-2-yl)-2,5diphenyltetrazolium bromide.

As shown in Fig. 2, DA inhibited the proliferation of MCF-7 cells in a dose- and time-dependent manner. Cell growth was suppressed by 98.2, 83.4 and 15.6% after treatment with 0.32, 0.08 and 0.02 mM DA, respectively. The rate of tumor cell growth inhibition with 0.32 mM DA was >90% and the IC_50_ values of the 24, 48 and 72 h time courses were 0.080±0.022, 0.050±0.016 and 0.029±0.050 mM, respectively.

### DA induces the apoptosis of MCF-7 cells

**Figure 3:**
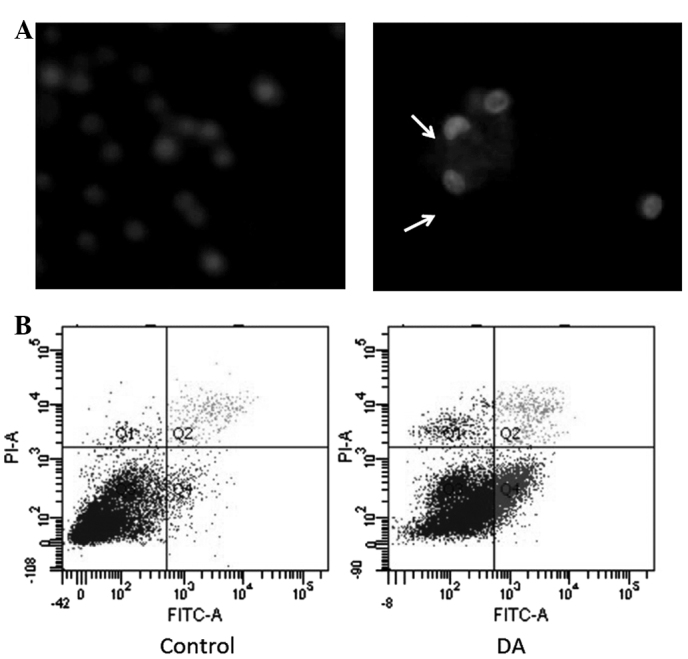
DA induced MCF-7 cell apoptosis. (A) MCF-7 cells treated with 0.05 mM DA or DMSO as a control for 48 h were subjected to DAPI staining to detect apoptotic nuclei. (B) MCF-7 cells were treated with 0.05 mM DA or DMSO as a control for 48 h, followed by an Annexin V-FITC assay. DA, β,β-dimethylacrylshikonin; DMSO, dimethyl sulfoxide; DAPI, 4,6-diamidino-2-phenylindole dihydrochloride hydrate.

DAPI nuclear staining was performed to detect morphological changes in DA-treated cells. DA-treated cells exhibited significant morphological changes, including nuclear condensation, DNA fragmentation and pronuclear apoptotic bodies (Fig. 3A). Annexin V/PI staining also demonstrated apoptosis, which was induced by DA (Fig. 3B). Overall, it was shown that DA visibly induced the apoptosis of MCF-7 cells in a dose-dependent manner.

### DA regulates the expression of apoptosis-related genes and proteins

The expression levels of Bcl-2, Bax and caspase-3 were detected by RT-PCR. Following incubation with DA for 48 h, the expression of Bax was noticeably enhanced in a dose-dependent manner, while Bcl-2 expression decreased according to the concentration of DA.

**Figure 4:**
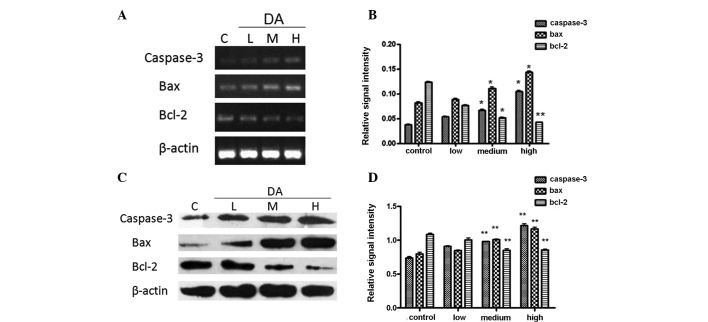
DA altered the expression of apoptosis-related genes in MCF-7 cells. (A and B) Effects of DA on the mRNA expression of Bcl-2, Bax and caspase-3 in MCF-7 cells. (B) Ratios of mRNA quantitation relative to β-actin. (C and D) Effects of DA on protein expression levels of apoptosis-related genes in MCF-7 cells. (D) Ratios of protein quantitation relative to β-actin. Each experiment was independently performed three times. ^*^P<0.05 and ^**^P<0.01, compared with the control group. DA, β,β-dimethylacrylshikonin; C, control; L, low DA concentration (0.0125 mM); M, medium DA concentration (0.025 mM); H, high DA concentration (0.05 mM).

The expression levels of apoptosis-related proteins, as detected by western blotting, were induced by DA in the same manner. The ratio of Bax/Bcl-2 protein expression was elevated markedly. Overall, it was observed that DA upregulated apoptosis-related genes (Fig. 4).

### DA regulates the activity of the nuclear factor (NF)-κB pathway

**Figure 5:**
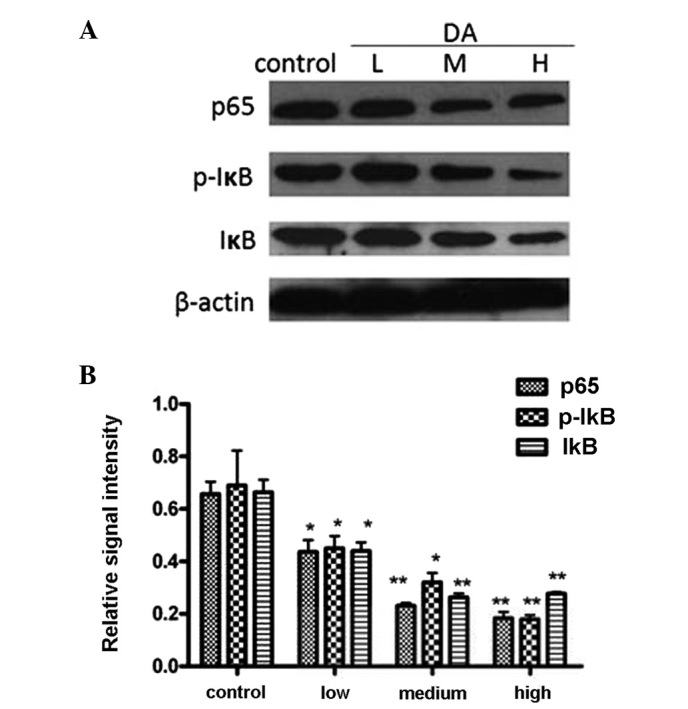
DA altered the activity of NF-κB pathways in MCF-7 cells. (A and B) Lysates from DA-treated cells were analyzed by western blotting for p65, IκB and p-IκB. (B) Ratios of protein quantitation relative to actin. Each experiment was independently performed three times. ^*^P<0.05 and ^**^P<0.01, compared with the control group. DA, β,β-dimethylacrylshikonin; NF-κB, nuclear factor κB; p-IκB, phosphorylated IκB; L, low DA concentration (0.0125 mM); M, medium DA concentration (0.025 mM); H, high DA concentration (0.05 mM).

To investigate the potential mechanisms involved in DA-induced apoptosis in MCF-7 cells, three important proteins involved in the NF-κB pathway, p65, IκB and phosphorylated-IκB were analyzed by western blotting. The results, as shown in Fig. 5, showed significant alterations in the expression of phosphorylated-IκB and p65, while the expression of IκB showed marginal change. This indicated that DA induced MCF-7 cell apoptosis by inactivating the NF-κB pathway.

## Discussion

There has been growing interest in using naturally occurring compounds to treat cancer. Shikonin, a compound isolated from the TCM ‘Zicao’, has been used as an ointment for wound healing. It has been demonstrated to inhibit the proliferation of certain types of cancer cells and to induce apoptosis through multiple signal transduction pathways (13,14). Its antitumor effects were first shown by its activity against S-180 tumor ascites at a dose of 5–10 mg/kg/day (15). It was also reported that the administration of shikonin reduced the volume of intestinal neoplasms induced by azoxymethane (16). After that, a number of other studies also demonstrated shikonin’s potential anticancer activities in several human tumors through inhibiting cancer cell growth, inducing apoptosis (6), inhibiting DNA topoisomerase I/II activity (17), anti-telomerase activity (18) and antiangiogenesis (5). However, its poor solubility and toxicity have significantly hampered its clinical use (19). Over the past few years, studies have been conducted with the aim of identifing novel shikonin derivatives with little toxicity. DA, a shikonin derivative, has been shown to have little toxicity, making it a promising anticancer agent (12). However the anticancer effects and mechanisms of DA in human breast cancer cells have not been elucidated. In the present study, the anticancer effects and mechanisms of DA in MCF-7 cells were investigated *in vitro* and it was observed that DA was able to suppress the proliferation of MCF-7 cells and induce cellular apoptosis time and dose dependently.

Previous studies have demonstrated that shikonin-like compounds cause cell apoptosis through the activation of a caspase-dependent pathway in numerous types of cancer cells. The treatment of chronic myelogenous leukemia K562 cells (5), human prostate cancer PC-3 cells (20), melanoma cells (6) and osteosarcoma cells (21) with shikonin induced apoptosis through increased caspase-3 activity. Our previous studies also showed that DA induced apoptosis in hepatocellular carcionoma SMMC-7721 cells (7) and gastric cancer SGC-7901 cells (22). To further investigate the underlying mechanisms of its antiproliferative effects, the present study investigated the expression of apoptosis-related proteins and the activity of the NF-κB pathway in DA-treated MCF-7 cells.

Apoptosis, the stereotypic program of cellular suicide regulated by a variety of factors, is critical in tumorigenesis and tumor progression (23). Studies have shown that shikonin-like compounds are able to induce apoptosis in multiple cancer cells through various signal transduction pathways (9,10,12–15). It was observed that following treatment with DA, MCF-7 cells exhibited typical morphological apoptotic changes. Apoptosis is a complex process involving a variety of molecules. The mitochondrial-mediated signal transduction pathway is central in the regulation of apoptosis. Members of the Bcl-2 family are the key regulators of mitochondrial response to apoptotic signals, with individual members promoting or suppressing apoptosis. Bcl-2, an anti-apoptotic factor, negatively regulates this cellular suicide machinery, whereas another Bcl-2-homologous protein, Bax, promotes cell death by competing with Bcl-2 (24,25). To determine whether apoptosis-related genes contribute to the inhibitory effects of DA on MCF-7 cells, we measured the relative Bcl-2 and Bax expression levels. It was noted that DA decreased the expression of Bcl-2 but increased the expression of Bax in a dose-dependent manner. Moreover, caspase-3 expression was also upregulated by DA.

It was also observed that DA suppressed the activity of NF-κB, an important transcription factor in the regulation of the genes governing apoptosis. NF-κB serves as a prosurvival agent similar to Bcl-2 in various circumstances (17). NF-κB complexes are mostly composed of two heterodimeric subunits of p50 and p65. In unstimulated cells, the NF-κB is in an inactive form within the cytosol, complexed to an inhibitory IκB-α protein (26,27). Following exposure to various carcinogens and growth stimuli, IκB-α may be phosphorylated and degraded by releasing the free NF-κB transcription factor. After the free NF-κB translocates into the nucleus, the genes with κB reporter regions in their promoters may be activated, the functions of which are closely associated with abnormal proliferation and survival of cancer cells (28). In the present study, it was observed that DA inactivated the NF-κB pathway through inhibiting the phosphorylation of IκB-α and downregulating p65 subunit expression.

In conclusion, DA was able to inhibit the proliferation and growth of MCF-7 cells *in vitro* by inducting apoptosis via the activation of caspase-3 and alteration of the apoptosis-related genes Bcl-2 and Bax. These alterations may be associated with inactivation of the NF-κB pathway through the downregulation of p65 and inhibition of IκB-α phosphorylation. These results suggest that DA has promise for potential clinical use as an anticancer agent in treating breast cancer. However, its mechanisms in other signaling pathways require discussion in further studies.
